# Neural Degeneration in the Retina of the Streptozotocin-Induced Type 1 Diabetes Model

**DOI:** 10.1155/2011/108328

**Published:** 2011-11-17

**Authors:** Yoko Ozawa, Toshihide Kurihara, Mariko Sasaki, Norimitsu Ban, Kenya Yuki, Shunsuke Kubota, Kazuo Tsubota

**Affiliations:** ^1^Laboratory of Retinal Cell Biology, Keio University School of Medicine, 35 Shinanomachi, Shinjuku-Ku, Tokyo 160-8582, Japan; ^2^Department of Ophthalmology, Keio University School of Medicine, 35 Shinanomachi, Shinjuku-Ku, Tokyo 160-8582, Japan

## Abstract

Diabetic retinopathy, a vision-threatening disease, has been regarded as a vascular disorder. However, impaired oscillatory potentials (OPs) in the electroretinogram (ERG) and visual dysfunction are recorded before severe vascular lesions appear. Here, we review the molecular mechanisms underlying the retinal neural degeneration observed in the streptozotocin-(STZ-) induced type 1 diabetes model. The renin-angiotensin system (RAS) and reactive oxygen species (ROS) both cause OP impairment and reduced levels of synaptophysin, a synaptic vesicle protein for neurotransmitter release, most likely through excessive protein degradation by the ubiquitin-proteasome system. ROS also decrease brain-derived neurotrophic factor (BDNF) and inner retinal neuronal cells. The influence of both RAS and ROS on synaptophysin suggests that RAS-ROS crosstalk occurs in the diabetic retina. Therefore, suppressors of RAS or ROS, such as angiotensin II type 1 receptor blockers or the antioxidant lutein, respectively, are potential candidates for neuroprotective and preventive therapies to improve the visual prognosis.

## 1. Introduction

Diabetic retinopathy, a vision-threatening disease, has long been regarded as a vascular disorder, which is staged clinically according to the proliferative status of the retinal vasculature [1]. The disorder involves hemorrhage, vascular obliteration, and the resulting neovascularization; these events subsequently cause fibrovascular proliferation and then retinal detachment, all of which can secondarily cause retinal neural degeneration. However, impaired visual function is recorded before the major vascular disorders appear [2]. Moreover, the atrophic appearance and dysfunction of the neural retina continue to worsen clinically after the vascular lesions become quiescent. Therefore, the diabetes-induced retinal neural degeneration may progress independently of the vascular lesions.

On the other hand, current treatments for diabetic retinopathy are mostly intended to regulate vascular changes mediated by the action of vascular endothelial growth factor (VEGF), by laser treatment to attenuate the hypoxic retinal cells that produce VEGF, and by anti-VEGF drugs, as well as blood glucose level. The future generation of therapies is expected to target neural tissue and to elicit a better visual prognosis. In the retina, a light stimulus activates the phototransduction pathway in the photoreceptor cells which span the outer layer of the retina, and is converted into electric signals which are then processed through the synaptic network system in the inner layer of the retina to finally transmit the signals to the brain to form visual function. This series of electric reactions in the retina can be recorded as an electroretinogram (ERG), which is used to evaluate retinal function objectively, both clinically in humans and experimentally in animals [2, 3]. Diabetes-related changes appear in the oscillatory potentials (OPs) in the ERG of human patients; these changes represent inner retinal dysfunction. OP changes in diabetic patients were first reported in the 1960s [2–4]. However, the molecular mechanisms underlying the diabetes-induced neural degeneration and dysfunction of the retina are still not well understood, and elucidating them is a current hot topic in the field of diabetes research. In this paper, we review the molecular mechanisms of neural degeneration revealed in the retina of the streptozotocin-(STZ-) induced type 1 diabetes models.

## 2. STZ-Induced Diabetes Models

Streptozotocin is a glucosamine-nitrosourea compound that was originally identified as an antibiotic, but is also known to have an anticancer effect. It is cytotoxic to pancreatic beta cells after being transported through glucose transporter 2 (GLUT2) [5] and therefore is used to generate type 1 diabetes model animals. Experimental diabetes can be induced in both rats and mice by the intraperitoneal injection of STZ [6–9]. In the mouse model, the blood glucose level reaches over 500 mg/dL after 1 month of diabetes, compared with about 100–120 mg/dL in control mice [6–9].

Although the above method is often used to make a diabetes model, there do not appear to be pathological neovascularization caused by vascular obliteration in these animals, which is typical finding in severe diabetic patients. However, this model shows apoptosis of the inner retinal neurons, such as ganglion cells and amacrine cells, and the activation of the Müller glial cells in the retina [10, 11], which can all contribute to the inner retinal dysfunction detected as OP changes in the ERG. Interestingly, VEGF, which is found at a high level in the diabetic retina [7, 12–15], is generally protective for neurons. Thus, the current treatments, which target the vascular impairments and VEGF, may not be effective for the neural pathogenesis. To develop new treatment approaches, it is therefore important to elucidate the molecular mechanisms underlying the neurodegenerative changes in diabetic retinopathy. For these studies, the STZ-induced model is useful, because it mimics the diabetes-induced OP change in the ERG observed in humans [6, 8, 16, 17]. 

In the following sections, we describe the molecular mechanisms of neural degeneration in the retina of the STZ-induced diabetes model, focusing on the renin-angiotensin system (RAS) [6, 7, 18, 19] and oxidative stress [8, 20, 21].

## 3. The RAS in the Neural Retina of Diabetes

The RAS was originally recognized as a regulator of the systemic blood pressure. One of its principal effectors, angiotensin II, was originally reported to be converted from a precursor peptide, angiotensinogen, which is produced in the liver and then sequentially converted into angiotensin II by enzymes, renin in the kidney and angiotensin-converting enzyme (ACE) in the lung, after it enters the circulation. Angiotensin II promotes vessel contraction to elevate the blood pressure, and this is known as systemic RAS. On the other hand, recent studies have revealed that all the RAS components can be generated within a given tissue or organ. This is called tissue RAS and is found in the heart, blood vessels, kidney, adrenal gland, pancreas, central nervous system, reproductive system, and lymphatic and adipose tissue [22], as well as the retina [23, 24]. Tissue RAS can be regulated independently of the systemic RAS [22].

Angiotensin II has long been known to have a role in diabetic complications, such as nephropathy and retinopathy, with evidence indicating that it affects the vascular system of each organ or tissue [7, 18, 25] via tissue RAS, in a paracrine fashion [25, 26]. Thus, the common mechanism of these complications has been believed to be the effects of angiotensin II on the vascular system [7, 18, 25]. However, recent studies using the STZ-induced diabetes model have begun to reveal the influences of angiotensin II on neural cells [6, 27].

### 3.1. Influence of the RAS on Synapses

**Figure 1:**
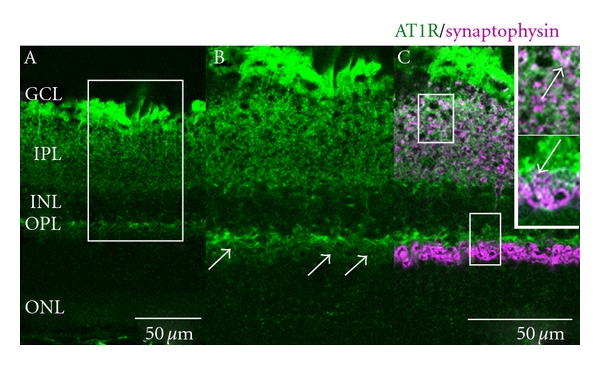
Expression of angiotensin II type 1 receptor and synaptophysin in the mouse retina. Expression of AT1R (green) in the normal retina (A–C). The boxed area in A is magnified in B and C (merged in C), and the boxed areas in C are magnified in the insets of C. AT1R is coexpressed with synaptophysin (pink), a presynaptic vesicle protein, in the IPL (arrow in the upper inset of C) and OPL (arrows in B and the lower inset of C). AT1R, angiotensin II type 1 receptor; GCL, ganglion cell layer; IPL, inner plexiform layer; INL, inner nuclear layer; OPL, outer plexiform layer; ONL, outer nuclear layer [28].

In STZ-induced diabetes model mice, angiotensin II and its type 1 receptor (AT1R) are upregulated in the retina [6, 7]. Interestingly, AT1R is abundantly coexpressed with synaptophysin, a synaptic vesicle protein critical for neurotransmitter release, in the inner layer of the normal mouse retina (Figure 1) [28], consistent with AT1R signaling role in modulating synaptic activity in the central nervous system [29].

Kurihara et al. analyzed the role of AT1R in the diabetic retina by administering the AT1R blockers telmisartan and valsartan to STZ-induced diabetes model mice [6]. AT1R blockers suppressed the OP change in this model that reflects impaired inner retinal function. Therefore, the visual functional impairment in this STZ-induced diabetes model is, at least in part, caused by AT1R signaling. Moreover, AT1R reduced synaptophysin expression in a posttranscriptional fashion in the retina of the diabetes model mice. This observation is consistent with the fact that the visual dysfunction in this model originates in the inner layer of the retina, where the synaptic network system is located.

### 3.2. Direct Influence of the RAS on Neurons

The molecular mechanism underlying the decrease in synaptophysin protein was analyzed using a neuronal cell line originated from a pheochromocytoma, PC12D. In this cell line, AT1R is coexpressed with synaptophysin which is posttranscriptionally decreased by adding angiotensin II to the culture medium [6]. This decrease is inhibited by both AT1R blockers and AT1R knockdown using shRNA. Downstream of AT1R, ERK is activated, which was also confirmed using inhibitors and shRNA. This posttranscriptional reduction of synaptophysin protein results from its excessive degradation through the ubiquitin proteasome system (UPS) [6]. Notably, this in vitro experiment showed that angiotensin II has a direct effect on neuronal cells. ERK is activated in the diabetic retina in vivo, suggesting that the same pathway is involved in the neuronal pathogenesis in the retina of the STZ-induced diabetes model. In another report on the effect of an ACE inhibitor in these model animals, it was concluded that the drug inhibited retinal degeneration by reducing the blood pressure and blood glucose level [30]. However, the in vivo results of Kurihara et al. were obtained under conditions in which the blood pressure and blood glucose levels did not change, further supporting the idea that RAS has a direct effect on neurons in vivo.

The role of angiotensin II in diabetic retinopathy has been studied clinically in the Diabetic Retinopathy Candesartan Trials (DIRECTs) [31, 32], in which the effect of an AT1R blocker on the incidence and progression of diabetic retinopathy was evaluated using the Early Treatment Diabetic Retinopathy Study (ETDRS) scale. Although this scale has been established to evaluate vascular lesions of diabetic retinopathy [33], the effect of the drug may also involve improving the retinal neural condition taking into account the data from the animal model [6].

### 3.3. RAS and UPS

Angiotensin II induces pathological UPS activity in various tissues, including vascular smooth muscle [34]. Angiotensin II promotes the expression of atrogin-1, an E3 ubiquitin ligase selective for skeletal muscle wasting [35]. Notably, another animal model of retinal tissue inflammation, endotoxin-induced uveitis and retinitis, in which RAS is also responsible for its neurodegenerative changes [28], shows the involvement of UPS-mediated excessive degradation of rhodopsin, a visual substance, in the neuronal dysfunction of the retina [36, 37]. Given that the RAS contributes to various kinds of diabetic complications, the UPS may have important roles in the diabetes-induced pathogenesis, a possibility meriting further study.

## 4. Oxidative Stress in the Neural Retina of Diabetes

Oxidative stress is another key modulator of diabetic complications [8, 20, 38]. Reactive oxygen species (ROS) can be produced in mitochondria through the electron transport chain [39, 40]. Keeping the levels of mitochondrial ROS at normal levels by pharmacological method prevents the activation of protein kinase C, the formation of advanced glycation end products, sorbitol accumulation, and NF-*κ*B activation, all of which are coupled with diabetes-induced vascular endothelial cell damage [40]. In this section, we introduce the mechanism of oxidative stress-induced neural degeneration observed in the retina of the STZ-induced diabetes model [8].

### 4.1. Influence of Oxidative Stress on Synapses

**Figure 2:**
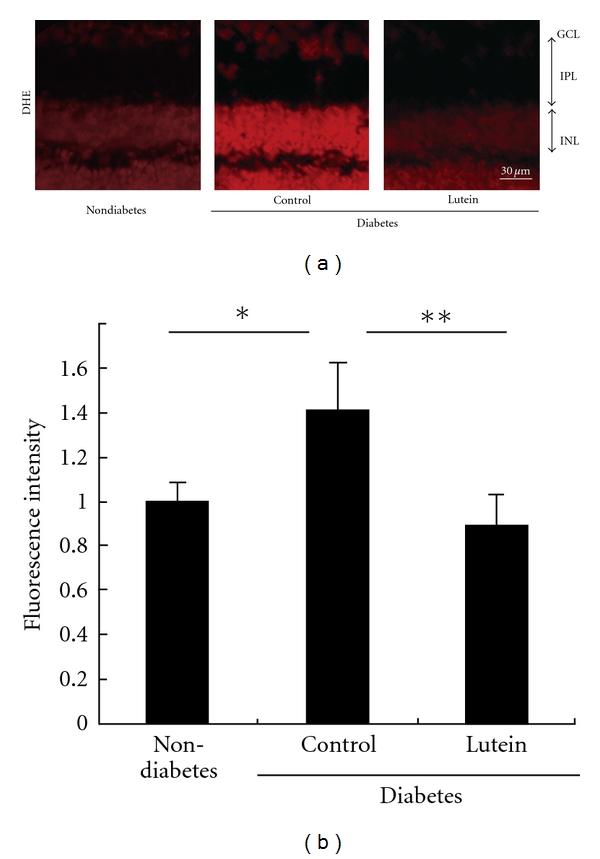
Oxidative stress in the retina of the STZ-induced diabetes model mouse. Dihydroethidium (DHE) indicates ROS in the retina (a). The level of diabetes-induced ROS in the retina is decreased by constant lutein treatment. Fluorescence intensity in the INL relative to that of nondiabetic mice was measured by the Image J program (b). DHE, dihydroethidium; ROS, reactive oxygen species; GCL, ganglion cell layer; IPL, inner plexiform layer; INL, inner nuclear layer (original copyright; [8], Figure 1, reproduced with the kind permission of Springer Science + Business Media.)

The retinal neuronal cells are influenced by ROS: in the STZ-induced diabetes model mice, both the decrease in synaptophysin protein and the ERG impairment are suppressed under the constant administration of an antioxidant, lutein, which suppresses the local ROS (Figure 2) and ERK activation in the diabetic retina [8]. This influence, shown by Sasaki et al., is observed as early as 1 month from diabetic onset, the same time as the RAS influence reported by Kurihara et al. Since lutein also did not change the blood glucose level in this analysis, but reduced the ROS level in the retina with diabetes, the effect of lutein in the retina was, at least in part, through the reduction of the diabetes-induced local ROS.

Another influence of ROS related to neuronal activity is exerted on the expression of brain-derived neurotrophic factor (BDNF). This factor regulates axonal growth and synaptic activity as well as neuronal survival [41]. The level of BDNF in the diabetic retina is decreased [8, 42], but lutein treatment prevents this reduction [8]. The BDNF level is regulated by neuronal synaptic activity [43], suggesting that the mechanism of its preservation in the retina of diabetic mice by lutein administration may involve the preservation of synaptophysin protein and the subsequent protection of neuronal synaptic activity.

In addition to promoting BDNF expression [43], synaptic activity promotes neuronal survival through depolarization of the cell membrane and increasing the level of intracellular calcium in the neuronal cells [44]. Therefore, the synaptic change observed in the diabetic retina affects, at least in part, retinal neuronal survival and visual function. Sasaki et al. showed that the thickness of the inner layer of the retina, including the retinal ganglion cells and amacrine cells, in the diabetes model mice is reduced 4 months after diabetes onset [8], consistent with the previous report showing similar changes in the model rats 7.5 months after diabetes onset [10]. However, in lutein-administered mice, in which the synaptophysin and BDNF levels are preserved after 1 month of diabetes, the thickness is preserved, and the neuronal cells survive by constant treatment [8]. Thus, the antioxidative treatment by lutein appears to protect the neuronal cells from apoptosis.

The antioxidant, lutein, is presently being studied for its preventive effects on the progression of age-related macular degeneration (AMD), a vision-threatening disease, as a micronutrient supplement. It is a yellow pigment and can filter blue light, which has a high energy level that is toxic to the retina. However, interestingly, the preventive effect of lutein observed in the retina of the STZ-induced diabetes model occurs by the ROS reduction rather than the filtering of light energy. Moreover, the fact that lutein is physiologically delivered to the retinal neurons [45] suggests that it might act directly in the retina, and not only as an antioxidant. Further studies aimed at elucidating whether lutein's effects involve pathways other than the antioxidative pathway should be performed.

### 4.2. Other Influences of Oxidative Stress in the Retina

The influence of diabetes on neural retinal cells has also been analyzed from other viewpoints. Under diabetic conditions, the increase in superoxide anions in the neural retina leads to a decreased bioavailability of nitric oxide which is originally induced in the retina for tissue protection; the decrease in nitric oxide action increases the formation of peroxynitrite, which inhibits the downstream signaling of a neuroprotective factor, nerve growth factor (NGF), and neuronal survival [46].

Müller glial cells, which contribute to the homeostasis of the retina, are also damaged by oxidative stress [47, 48]. These cells originate from a common retinal progenitor cell with neuronal cells and populate the neural retina. Therefore, the damage of these cells can also influence visual function. The influences of oxidative stress on Müller glial cells involve the downregulation of a potassium channel, Kir4.1, a water channel, aquaporin 4 [48], and matrix metalloproteinase-7 (MMP-7) which converts a toxic factor for neurons, pro-nerve growth factor (pro-NGF), to a neuroprotective factor, NGF [47]. Therefore, there are multiple pathways downstream of oxidative stress that can cause retinal neural cell damage and decrease visual function.

## 5. Crosstalk between Angiotensin II and ROS Signals

**Figure 3:**
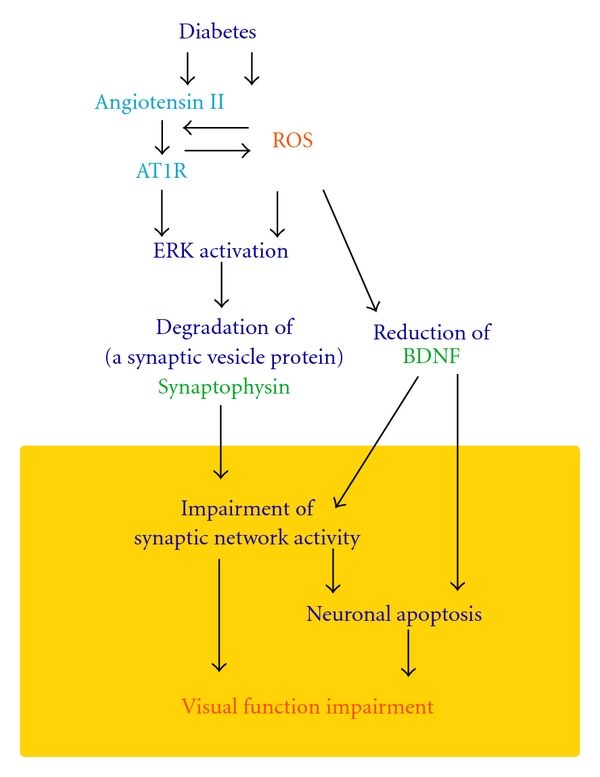
Model of retinal neural degeneration and visual impairment in the STZ-induced diabetes model mouse. Cross-talk between AT1R signaling and ROS is involved in the neural degeneration of the diabetic retina. Downstream of both these effectors, ERK activation reduces synaptophysin, while ROS also decrease BDNF. These changes impair visual function most probably through synaptic network abnormalities and neuronal apoptosis. AT1R, angiotensin II type 1 receptor; ROS, reactive oxygen species; BDNF, brain-derived neurotrophic factor.

Synaptophysin protein is decreased in the retina of the STZ-induced diabetes model through both the AT1R-ERK axis and the ROS-ERK axis, suggesting the involvement of cross talk between angiotensin II and ROS signals in the diabetic retina (Figure 3). Angiotensin II can activate nicotinamide adenine dinucleotide phosphate (NAD(P)H) oxidase via AT1R stimulation and produces ROS, as shown in the pathogenesis of atherosclerosis [49–51]. Moreover, angiotensin 1-7, which are cleaved from angiotensin II and act as its negative regulators, are reduced in the retina with diabetes [52], and their overexpression reduces diabetes-induced oxidative stress in the retina, suggesting that their reduction might also be related to the cross talk.

Indeed, the effect of angiotensin II on the retinal leukostasis in diabetes occurs at least partly through NAD(P)H oxidase-related ROS generation, as shown using apocynin, an NAD(P)H oxidase inhibitor, and *N*-acetylcysteine (NAC), an antioxidant [53]. The ROS generated downstream of AT1R further upregulate multiple inflammatory cytokines, such as TNF-*α*, IL1-*β*, and IFN-*γ*, which further induce ROS [51]. Collectively, these findings support the idea that RAS-ROS cross talk has a large role in the pathogenesis of the diabetes-induced degeneration of neural tissue. 

Another outcome of the cross talk in the diabetic retina may be the expression of VEGF. Müller glial cells are the responsible cells for VEGF induction in the diabetic retina, and this induction is regulated by hypoxia-inducible factor-1*α* (HIF-1*α*) [54], which can be activated by oxidative stress [55]. VEGF induction in the diabetic retina is also regulated by RAS [7], suggesting that cross talk may occur in the Müller glial cells.

Further studies are required to assess the contribution of the RAS-ROS interaction in the diabetic retinal degeneration that leads to impaired visual function and its possibility as a therapeutic target for this impairment.

## 6. Summary

Retinal neural cell loss is involved as one of the main causes for visual deficits in diabetes, and given that the retinal neural tissue eventually regulates diabetes-induced vascular disorders [54], it is essential to understand the molecular mechanisms underlying the pathology in the retinal neural cells to establish the next generation of therapies. To the best of our knowledge, at present the angiotensin II modifiers and antioxidants are the most promising candidates for future neuroprotective and preventive therapies aimed at improving neuronal cell survival and normalizing the microenvironment, such as cytokine production in the neural tissue, in the diabetic retina.
